# Subcortical white matter infarcts predict 1-year outcome of fatigue in stroke

**DOI:** 10.1186/s12883-014-0234-8

**Published:** 2014-12-12

**Authors:** Wai Kwong Tang, Yang Kun Chen, Hua Jun Liang, Winnie Chiu Wing Chu, Vincent Chung Tony Mok, Gabor S Ungvari, Ka Sing Wong

**Affiliations:** Department of Psychiatry, Chinese University of Hong Kong, Hong Kong, SAR China; Department of Neurology, Dongguan People’s Hospital, Dongguan, Guangdong PR China; Department of Imaging and Interventional Radiology, Chinese University of Hong Kong, Hong Kong, SAR China; Department of Medicine and Therapeutics, Chinese University of Hong Kong, Hong Kong, SAR China; The University of Notre Dame Australia/Marian Centre, Perth, Australia; School of Psychiatry and Clinical Neurosciences, University of Western Australia, Perth, Australia; Department of Psychiatry, Shatin Hospital, Shatin, NT, Hong Kong, SAR China

**Keywords:** Fatigue, Stroke, Infarcts, MRI, Outcome

## Abstract

**Background:**

Fatigue is common in stroke survivors. Lesion location may influence the risk of poststroke fatigue (PSF) but it is uncertain whether location has an impact on the prognosis of PSF. This study examined the association between PSF outcome and infarct location.

**Methods:**

The study sample comprised 435 Chinese patients with acute ischemic stroke admitted to the acute stroke unit of a university affiliated regional hospital in Hong Kong. Three and fifteen months after the onset of the index stroke a research assistant administered the Fatigue Severity Scale (FSS). PSF was defined as a FSS score of 4.0 or above. Of the 139 patients with PSF three months poststroke, 97 (69.8%) attended the 15-month follow-up, when 50 (51.5%) patients still had PSF (‘non-remitters’) and 47 (48.5%) did not report fatigue (‘remitters’). The presence and location of infarcts were evaluated with magnetic resonance imaging.

**Results:**

In comparison with the remitters, the non-remitters were more likely to have subcortical white matter infarcts (40.0% vs 21.3%, p = 0.046). These infarcts remained an independent predictor of non-remission of PSF in the multivariate analysis, with an odds ratio of 4.208 (p = 0.011).

**Conclusions:**

The results suggest that subcortical white matter infarcts may influence the outcome of PSF. Further investigations are needed to explore whether infarcts have any impact on the response of PSF to pharmacological or psychological interventions.

## Background

Fatigue is very common following stroke, with a reported frequency of 35% to 92% [1]. One year after the index stroke, the frequency of poststroke fatigue (PSF) varies from 18% to 36% [2-4]. Only a few studies have examined the possible clinical predictors of non-remission of PSF. Snaphaan et al. [5] followed up 108 stroke survivors for 1.5 years and found no significant differences between patients with PSF and patients who recovered. Factors associated with PSF 1 year poststroke (incident or persistent) include younger [3] or older age, [2] female sex [2], more severe stroke [6], poor functional status [7], fatigue severity at baseline [3], insomnia [6] and depressive symptoms [2-4,6].

The impact of lesion location on PSF at the chronic stage of stroke remains controversial. The abovementioned follow-up study [5] found no association between PSF at 1.5 years poststroke and lesion location (cortical vs subcortical or subcortical white matter vs basal ganglia vs thalamus) or white matter hyperintensities. No association was reported between stroke type [2,6], laterality [2,8] and site [4] and PSF at 1 year poststroke. None of these studies compared remitted and non-remitted PSF patients at follow up, or thoroughly evaluated lesion location [2,6,8] or used validated measures of PSF [6].

It has been suggested that structures involved in the subcortical attentional network play a critical role in the pathophysiology of PSF [3,9]. Several studies reported associations between lesion location and PSF with right insular [10], infratentorial [5], brainstem [11], internal capsule and basal ganglia strokes [12], although there were negative reports [13,14]. If infarct locations affect the risk of PSF, then it is possible that they also influence its outcome.

As no data have been published on the brain imaging predictors of outcome in PSF, the aim of this study was to determine the relationship between infarct location and the course of PSF.

## Methods

### Patients

All included patients attended the 3- and 15-month follow ups in person. A total of 4,048 patients with first-ever or recurrent acute ischemic stroke were admitted to the Acute Stroke Unit of the Prince of Wales Hospital between May 2007 and March 2011. Prince of Wales is a university-affiliated general hospital serving a population of 800,000 in Hong Kong. Of the 4,048 patients, 1,534 received an MRI examination; 710 patients did not attend the 3-month follow-up because they could not be contacted (n = 328), refused to participate (n = 227), were physically frail (n = 109) or were deceased (n = 46). The study sample comprised 435 (28.4%) patients who fulfilled the entry criteria listed below. Excluded patients (n = 1099) were older (67.7 ± 12.4 vs 65.9 ± 9.9, p =0.008) and had a higher National Institutes of Health Stroke Scale (NIHSS) [15] scores (5.2 ± 5.3 vs 3.7 ± 3.2, p < 0.001) but the sex distribution of the two groups was close to even (44.5% vs 41.1% female, p = 0.233).

The inclusion criteria for the study were 1. Chinese ethnicity; 2. Cantonese as the primary language; 3. age of 18 or above; 4. well-documented (clinical presentation and CT scan of the brain) first or recurrent acute stroke occurring up to 7 days before admission; and 5. ability and willingness to give consent. The exclusion criteria included 1. transient ischemic attack, cerebral haemorrhage, subdural haematoma or subarachnoid haemorrhage; 2. another stroke before the 3-month follow up; 3. history of a CNS disease such as tumour, Parkinson’s disease, etc.; 4. history of depression or substance abuse/dependence or other psychiatric disorder before the index stroke; 5. moderate or severe dementia defined as a Mini-Mental State Examination (MMSE) [16] score less than 20; and 6. severe aphasia and hearing or visual impairment.

Of the 435 patients screened, 139 reported PSF at the 3-month follow-up (baseline). One-year after the baseline assessment (i.e., 15 months after the index stroke), 97 (69.8%) of these 139 patients attended the follow-up assessment. Patients who did not attend the follow-up (n = 42) were older (68.3 ± 9.2 vs 63.5 ± 10.0, *p* = 0.008) than those who attended the follow-up. The sex distribution (40.5% vs 52.6% female, *p* = 0.190), mean age (65.7 ± 11.0 vs 67.2 ± 10.5; p = 0.319), and mean NIHSS (3.9 ± 3.4 vs 3.9 ± 2.9, *p* = 0.978) score of the two groups were similar. The study protocol was approved by the Clinical Research Ethics Committee of the Chinese University of Hong Kong. All participants signed a consent form.

### Collection of demographic and clinical data

A research nurse collected the demographic data (age, sex and years of education), history of hypertension, diabetes mellitus, hyperlipidemia and previous stroke, and assessed the stroke severity using the NIHSS [17] on admission. At the 15-month follow-up, recurrence of stroke was also recorded.

### Assessment of PSF

Three and 15 months after the onset of the index stroke, a trained research assistant who was blind to the patients’ radiological data administered the Chinese version of the Fatigue Severity Scale (FSS) [18,19]. PSF was defined as an FSS score of 4.0 or more. Patients who had PSF at the 3- but not 15-month follow-up constituted the ‘remitters’ group.

Three months after the index stroke, global cognitive functions were evaluated with the Cantonese version of the MMSE [16]. Insomnia was assessed by the following question: ‘*How many days of insomnia per week do you think you have?* [20,21]’. Patients were identified as having insomnia if they scored more than 0 days. Depressive symptoms were measured with the locally validated Geriatric Depression Scale (GDS) [22]. The Barthel Index (BI) [23] was used to assess the extent of patients’ disability in daily functions. Patients were also asked if they experienced pain in the last month with the following question: ‘*Do you consider yourself as having pain?’*

### Radiological examination

MRI was performed with a 1.5-T system (Sonata, Siemens Medical, Erlangen, Germany) within 7 days of admission. The following sequences were acquired: diffusion weighted imaging (DWI) (TR/TE/excitation = 180/122/4, matrix = 128 × 128, FOV = 230 mm, slice thickness/gap = 5 mm/1 mm, echo planar imaging, single shot factor = 90) with three orthogonally applied diffusion gradients (b values of 1000 500, and 0 s/mm2); and axial spin echo (SE) T1-weighted FFE sequence (TR/TE/excitation = 25/2.3/1, flip angle 30° FOV = 230 mm, slice thickness/gap = 3 mm/-1.5 mm, matrix = 256 × 256), turbo spin echo (TSE) T2–weighted (TR/TE/excitation = 2622/80/2, turbo factor of 18, FOV = 230 mm, slice thickness/gap = 5 mm/0.5 mm, matrix = 512 × 346); axial Flair (fluid attenuated inversion recovery) sequence (TR/TE/TI/excitation = 11000/125/2800/1, turbo factor of 31, FOV = 230 mm, slice thickness/gap = 5 mm/0.5 mm, matrix = 352 × 248) and blood sensitive gradient echo sequence (TR/TE/excitation = 350/30/2, a flip angle of 30 degrees, slice thickness/gap = 5 mm/0.5 mm, FOV = 230 mm, matrix = 256 × 256).

A neurologist (YKC) who was blind to the psychiatric diagnoses and clinical data other than age and sex assessed the MRIs for the following.

1. Infarcts; the total area of acute infarcts on DWI was measured using manual outlines. Acute infarcts were defined as areas of restricted water diffusion identified on DWI with b values of 1000 together with hypointensity on the corresponding ADC map. Acute infarcts were divided into cortical (frontal, temporal, parietal and occipital), subcortical (subcortical white matter, basal ganglia and thalamus) and infratentorial (brainstem and cerebellum). If an infarct involved both subcortical white and grey matter, it was counted as both a subcortical white matter infarct and a grey matter infarct. The total volume was calculated by multiplying the total area by the sum of the slice thickness and the gap. The number of old infarcts was also recorded. Chronic infarcts were defined as focal hyperintensities on T2-weighted and hypointensities on T1-weighted images with size ≥3 mm. Lesions less than 3 mm were considered as dilated perivascular space. Intra-rater reliability tests were performed on 20 patients; the kappa values for the volume and number of infarcts were 0.96 and 0.94, respectively.
2. The severity of WMHs was graded using the Fazekas scale at the periventricular and deep white matter regions, respectively, with a scale ranging from 0, 1, 2, to 3 [24].

### Statistical analysis

All statistical tests were performed using SPSS for Windows (Release 20.0; SPSS Inc., Chicago, IL). The demographic and clinical variables and radiological characteristics of the remitters were compared with those of the non-remitters using Fisher’s exact test and Student’s t-test, as appropriate. Risk factors with values of p < 0.10 were then analysed with multivariate logistic regression analysis employing a forward stepwise selection strategy. If the correlations between any of these putative risk factors were >/=0.50, then additional models were examined to rule out co-linearity. In the analysis, the odds ratio of any independent risk factor was interpreted as the risk of non-remission of PSD when all other risk factors were held constant. The level of significance was set at 0.05.

## Results

The final sample (n = 97) had the following characteristics: 51 patients (55.6%) were women, and 12 (12.4%), 60 (70.1%), 30 (30.9%), 54 (55.7%) had histories of stroke, hypertension, diabetes mellitus and hyperlipidemia, respectively. The mean age and education (in years) were 65.0 ± 10.0 and 6.0 ± 4.9, respectively. The mean NIHSS score on admission was 3.9 ± 3.1. The BI, MMSE, GDS and FSS scores at baseline were 19.1 ± 2.4, 27.2 ± 2.6, 6.4 ± 4.4, and 5.0 ± 0.8, respectively. Forty-four (45.4%) patients experienced pain and 65 (67.0%) reported insomnia. Three (3.1%) patients had another stroke before the 15-month follow-up.

Of the 97 patients who attended the 15-month follow-up, 50 (51.5%) still had PSF (non-remitters) and 47 (48.5%) did not (remitters). Compared with the remitters, the non-remitters had higher baseline GDS scores (p = 0.002), were more likely to report pain (p = 0.001) and experienced more insomnia (Table 1). The non-remitters were more likely to have subcortical white matter infarcts (40.0% vs 21.3%, p = 0.046) (Table 2). There was no significant association between baseline FSS score and the presence of subcortical white matter infarcts (Spearman’s rho = −0.061, p = 0.504). The medians of the difference between baseline and follow-up FSS scores in patients with and without subcortical white matter infarcts were 0 and −1, respectively (Mann–Whitney U test, p < 0.001).

**Table 1:** **Demographic and clinical characteristics of non-remitters and remitters of poststroke fatigue**

	**Non-remitters**	**Remitters**	
	**N = 50**	**N = 47**	
	**Mean ± SD**	**Mean ± SD**	***p***
Age	62.7 ± 9.9	64.4 ± 10.1	0.421^a^
Female	30 (60.0)	21 (44.7)	0.131^b^
Education (years)	5.6 ± 4.1	6.4 ± 4.8	0.389^a^
Hypertension	36 (72.0)	32 (68.1)	0.674^b^
Diabetes mellitus	19 (38.0)	11 (23.4)	0.120^b^
Hyperlipidemia	30 (60)	24 (55.1)	0.376^b^
Previous stroke	8 (16.0)	4 (8.5)	0.263^b^
NIHSS total score	4.1 ± 2.2	3.7 ± 3.5	0.464^a^
Barthel index	19.1 ± 2.4	19.0 ± 2.7	0.783^a^
Mini-mental state examination score	27.0 ± 2.4	27.8 ± 2.6	0.134^a^
Geriatric depression scale score	7.6 ± 4.4	4.9 ± 3.9	0.002^a^
Fatigue severity scale score	5.1 ± 0.6	5.0 ± 1.1	0.564^a^
Presence of pain	31 (63.3)	13 (27.7)	0.001^b^
Presence of insomnia	39 (79.6)	26 (55.3)	0.011^b^
Recurrent stroke	2 (4.0)	1 (2.1)	1.000^C^

NIHSS indicates National Institutes of Health Stroke Scale.

^a^t test; ^b^Chi-square test; ^C^Fisher’s exact test.

**Table 2:** **Radiological characteristics of non-remitters and remitters of poststroke fatigue**

**Variable**	**Non-remitters**	**Remitters**	
	**N = 50**	**N = 47**	
	**Mean ± SD**	**Mean ± SD**	***p***
Number of acute infarcts	2.1 ± 2.9	1.1 ± 1.2	0.131^C^
Volume of acute infarct (cm^3^)	1.6 ± 2.1	3.1 ± 5.3	0.777^C^
Cortical	10 (20.0)	10 (21.3)	0.877^b^
Frontal	9 (18.0%)	4 (8.5%)	0.170^a^
Temporal	1 (2.0%)	1 (2.1%)	1.000^b^
Parietal	3 (6.0%)	2 (4.3%)	1.000^b^
Occipital	0 (0%)	2 (4.3%)	0.232^b^
Subcortical white matter	20 (40.0%)	10 (21.3%)	0.046^a^
Basal ganglia	8 (16.0%)	9 (19.1%)	0.684^a^
Thalamus	4 (8.0%)	3 (6.4%)	1.000^b^
Infratentorial	4 (8.0%)	9 (19.1%)	0.107^b^
Brain stem	4 (8.0%)	6 (12.8%)	0.516^b^
Cerebellum	1 (2.0%)	4 (8.5%)	0.195^b^
Fazekas DWMH score	1.1 ± 0.9	1.2 ± 0.8	0.549^C^
Fazekas PVH score	1.2 ± 0.8	1.3 ± 0.7	0.637^C^
Number of old infarcts	0.5 ± 1.1	0.7 ± 1.5	0.370^C^

DWMH indicates deep white matter hyperintensities; PVH, Periventricular hyperintensities.

^a^Chi-square test; ^b^Fisher’s exact test; ^C^Mann-Whitney U test.

The presence of subcortical white matter infarcts, pain, insomnia, age, sex, GDS and NIHSS score were entered in the regression model. The presence of subcortical white matter infarcts was a significant predictor of non-remission of PSD with an odds ratio of 4.208 (p = 0.011). Other predictors included pain and GDS score (Table 3).

**Table 3:** **Multivariate logistic model of the clinical determinants of non-remission of poststroke fatigue**

**Parameter**	**Adjusted odds ratio (95% C.I.)**	***p***
Subcortical white matter infarct	4.208 (1.396 -12.681)	0.011
Presence of pain	5.664 (2.102 - 15.268)	0.001
Geriatric depression scale score	1.218 (1.072 - 1.383)	0.002
Presence of insomnia		0.091
Age		0.660
Sex		0.731
NIHSS		0.329
R square		0.354

The presence of subcortical white matter infarcts, pain, insomnia, Geriatric Depression Scale score, age, sex and National Institute of Health Stroke Scale score were entered in the regression model.

## Discussion

To the best of our knowledge, this is the first report of an association between the location of acute infarcts and PSF outcome. The main finding is that subcortical white matter infarcts are associated with persistent fatigue 15 months after the index stroke in patients with well-established stroke.

A few studies have evaluated the possible impact of lesion location on PSF at the chronic stage of stroke, with negative results. Schepers et al. [25] did not find any association between laterality or type of stroke (ischemic vs haemorrhagic) and PSF at 1 year poststroke in 167 stroke patients [2]. Another study on 253 patients also found that stroke type was not related PSF at 1 year poststroke [6]. No association was found between PSF at 1.5 years poststroke and lesion location or white matter hyperintensities [3], and no relationship was found between lesion site and PSF 1 year poststroke in 99 stroke survivors [4]. Type or laterality of stroke was not related to increased fatigue in 242 patients at 6 months poststroke [8].

The importance of white matter lesions in the pathophysiology of fatigue has been reported in non-stroke patient populations. Reduced white matter volume was observed in chronic fatigue syndrome [26]. Increased white matter hyperintensities were also found to be associated with increased fatigue in systemic lupus erythematosus [27]. White matter micro-structural damage was correlated with fatigue severity in multiple sclerosis [28]. Our previous MRI study demonstrated that internal capsule infarcts were more common in PSF [12]. Alterations of the integrity of white matter were related to the outcome of late-life depression [29,30]. It is possible that by disrupting the subcortical attentional network subcortical white matter infarcts adversely affect the outcome of PSF in stroke.

The remission rate of PSF in this study is higher than the figures of 18-36% reported in the literature [2-4,7,8]. Ten of the 38 patients with PSF at baseline had recovered by the 1.5-year follow-up, yielding a remission rate of 26.3%; no predictor of remission could be found [5]. In another study, 10 of 86 patients with PSF at baseline recovered by follow-up, yielding a remission rate of 19.8%; again, remission could not be predicted [2]. The remission rate of PSF between 6–12 and 24 months poststroke was 22.7% [31] and 35.6% [7], respectively. A 6-month follow-up of 242 patients reported that 17.8% had significant reductions in fatigue [8]. Patients with a history of depression were excluded from our study, which may have reduced the proportion of patients with persistent fatigue because depressive symptoms were one of the predictors of non remission. Depressive symptoms also predicted PSF at follow up [2-4,32].

In this study, depression severity and the presence of pain and insomnia predicted the persistence of PSF. Pain and sleep disturbances have been found to be associated with PSF at baseline [33] and one-year follow up [6]. Depressive symptoms at baseline also predicted PSF at 1 to 1.5 year follow ups [5,25,31,34]. There is a symptomatic overlap between PSF and poststroke depression [35]. Fatigue can be a symptom [36] as well as a risk factor of PSD [37]. Fatigue can also occur in the absence of depression: only 28-38% of stroke patients with severe fatigue have depression [13,38]. Factor analysis has revealed that PSF and PSD can be separated into two distinct constructs [39]. Impairment of locomotion, rather than depressive symptoms, explained most of the variance of fatigue in one study of stroke patients [38]. Antidepressant treatment usually improves depression but not fatigue in patients with PSF [40]. Hence, PSF and poststroke depression should be treated as two distinct, albeit partially overlapping conditions.

### Limitations

The main limitation of this study is its relatively high loss of patients to follow-up (the ideal sample size would have been 653 if there had been no attrition) and its small sample size, which reduced its statistical power. Stroke severity in the final study sample was mild and patients who could not give consent due to dementia or aphasia were excluded. This selection bias may limit the generalizability of the findings to patients with more severe stroke.

## Conclusions

In conclusion, the findings indicate that subcortical white matter infarcts are associated with a higher risk of non-remission of PSF. Further investigations are needed to clarify whether infarcts have any impact on the response of PSF to pharmacological and psychological interventions.
